# 

**Published:** 1895-08

**Authors:** 


Vol. XLIX. AUGUST, 1895. No. 8.

LIBRARY
Indexed
S. G. O.

### THE DENTAL REGISTER

A MONTHLY JOURNAL.

DEVOTED TO THE INTERESTS OF THE PROFESSION.

EDITED BY
J. TAFT. D.D.S.
ASSOCIATE EDITORS,
WILL. H. WHITSLAR, D.D.S. N. S. HOFF, D.D.S.

PUBLISHED BY
SAM'L A. CROCKER & CO.,
SUCCESSORS TO SPENCER & CROCKER,
OHIO DENTAL AND SURGICAL DEPOT,
Nos. 117, 119, 121 WEST FIFTH STREET, CINCINNATI, OHIO.

**TERMS:—$2.00 per Annum, in Advance. Single Copies, 25 cts.**

